# Improving Involvement of Families of Small Babies through Family Education, Family Integration, and Multidisciplinary Teamwork: A Quality Improvement Initiative

**DOI:** 10.1097/pq9.0000000000000828

**Published:** 2025-07-30

**Authors:** Kris Nicole D. Mendoza, Hyacinth Lewis, Lynsey Garver, Laura Koegst, Elaine Kong, Molly Roberts, Jean Shirley, Devin Stoklosa, Christina Tryon, Tricia White, Colby Day

**Affiliations:** From the Division of Neonatology, Department of Pediatrics, University of Rochester, Rochester, N.Y.

## Abstract

**Introduction:**

Partnering with and educating families in the neonatal intensive care unit (NICU) is critical for infant neurodevelopment, parent wellness, and family support. Early family integration in care improves both short-term and long-term outcomes. This quality improvement project has 2 specific aims: (1) increase the percentage of small babies (born at <28 wk of gestation or <1000 g) whose families participated in a multidisciplinary family-centered care conference (FCCC) from 0% to 50%, and (2) increase family attendance at the first NICU follow-up clinic from 74% to 90%.

**Methods:**

Using the model for improvement, we conducted plan-do-study-act cycles with iterative interventions to achieve our aims. The FCCCs focused on promoting family involvement at the bedside, infant neurodevelopment, skin-to-skin care, family support, and transitions within the NICU and after discharge. Outcome, process, and balancing measures were tracked and analyzed for special cause variation using statistical process control charts.

**Results:**

Within 18 months, the percentage of infants whose families participated in an FCCC increased from 0% to 39% (48/123). Based on the post-FCCC survey, families found the FCCC helpful in providing information on how they can be involved in their infant’s care. They recommended the FCCC to other families, and they expressed interest in a second FCCC focused on preparing for the transition home.

**Conclusions:**

Family participation in a multidisciplinary FCCC increased over time but has not yet achieved the stated goal. We anticipate that further plan-do-study-act cycles will improve adherence to a robust FCCC program by integrating families into their infants’ care during critical developmental stages.

## INTRODUCTION

Parents of neonates admitted to the neonatal intensive care unit (NICU) experience heightened distress, anxiety, depression, and trauma symptoms.^1^ A parent’s psychological experience correlates with the quality of parental attachment and affects the parent–infant relationship and the infant’s behavioral and developmental outcomes.^1^ For very preterm infants, the parent–child relationship begins in the NICU, shaped by parents’ presence, bonding, holding, and responsiveness to their infant’s needs.^2^

Families play a vital role in the health outcomes of their infants, both as caregivers and decision-makers.^3^ Embracing families as decision-making partners and collaborators in their infant’s care has long been recognized as an optimal way of caring for babies in the NICU.^4^ Parents can also help improve the infant’s ability to cope with NICU stressors by providing appropriate, meaningful sensory stimuli and human contact.^2^ Maternal NICU involvement is associated with superior cognitive and language outcomes in early childhood.^5^ Skin-to-skin care (SSC) is related to decreased acute pain responses, improved weight gain, reduced hypothermia, earlier discharge, better cognitive outcomes in childhood, and enhanced nurturing and parent–child interactions.^6^ It has also been shown to impact mothers’ sense of competence and meaning, as well as improve attachment, mastery, and self-esteem.^2,7^

Many discussions with families in the NICU arise from a serious change in an infant’s clinical condition that requires medical decision-making, such as acute life-threatening situations, surgical interventions, establishment of long-term goals, or care redirection. However, many NICUs lack dedicated time for families to receive information on improving parental attachment and bonding activities, such as direct involvement in bedside care, SSC, 2-person care, oral immune therapy, and positive auditory stimulation. This gap in family-centered care prompted the authors to introduce a dedicated family-centered care conference (FCCC), aiming to improve both infant and family outcomes in the long run. We also anticipated that improving family integration in the NICU would result in improved follow-up rates at the initial NICU follow-up clinic (NFC) visit, as the FCCC emphasizes the importance of neurodevelopment.

## METHODS

### Context

The NICU at Golisano Children’s Hospital at the University of Rochester Medical Center serves as the regional perinatal center for more than 17 referring hospitals in a 9-county region of upstate New York. The NICU is a 68-bed, level IV unit with primarily single-family rooms. Yearly, there are approximately 3,500 deliveries, 1,100 NICU admissions, and 70–100 small baby admissions (defined as infants born less than 28 weeks gestation or weighing <1000 g).

Families were eligible to participate in FCCCs if they had infants born less than 28 weeks of gestational age or birthweight less than 1000 g. Families were not eligible if their child had a lethal congenital medical condition.

The University of Rochester institutional review board determined that this project was not human research, as defined by the Department of Health and Human Services and United States Food and Drug Administration regulations (study ID: STUDY 00007592, September 21, 2022).

### Team

The multidisciplinary NICU team includes a child life specialist, music therapist, assistant nurse manager, nurse-parent educator, family advisor, lactation medicine consultants, social workers, occupational therapists, neonatology fellows, and a neonatologist. Team members were selected to represent diversity in expertise in neonatal care, family support and education, and unit practices.

### Interventions

**Fig. 1.**
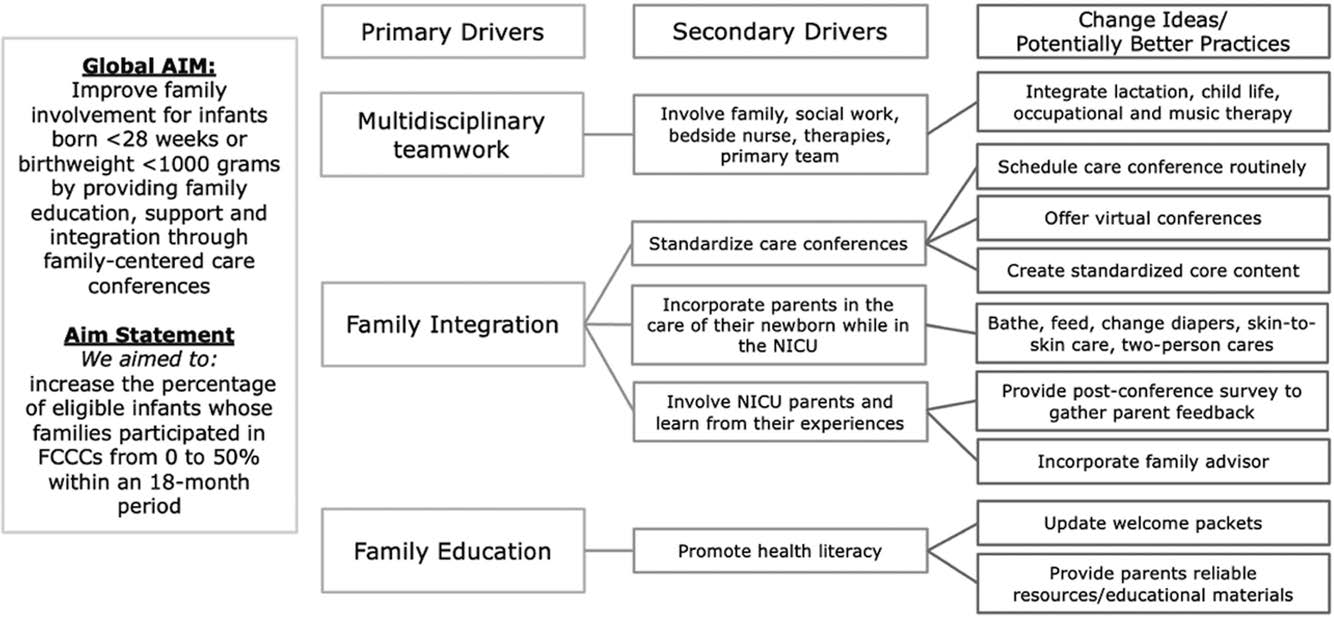
Key driver diagram outlining the key drivers and change ideas identified by the QI team to improve family involvement within the NICU.

The project used the Institute for Healthcare Improvement’s Model for Improvement^8^ as the framework, applying plan-do-study-act (PDSA) cycles to test interventions (Table 1). The team developed the key driver diagram (Fig. 1) and provided targeted education to faculty and staff regarding the FCCCs before initiation (PDSA no. 1, May 2022). The project leader introduced the concept of FCCCs to eligible families between the third and seventh day of their infant’s life. Depending on the patient’s clinical stability and team availability, conferences are scheduled between the first and fourth weeks of the infant’s life, allowing parents time to recover from the delivery and transition through the initial NICU course. In October 2022, the team initiated FCCCs using a standardized workflow (PDSA no. 2). We organized 60-minute conferences to allow all team members to discuss their areas of expertise. (**See table 1, Supplemental Digital Content 1**, which demonstrates team member roles and aspects of family involvement discussed during FCCCs, https://links.lww.com/PQ9/A690.) The conferences primarily focus on supporting the infant’s neurosensory and neuromotor development by encouraging families to be hands-on at the bedside and provide positive auditory experiences, SSC, 2-person care, and oral immune therapy. The team also discusses ways to enhance the NICU stay, including promoting communication among families, developmental specialists and staff, as well as supporting families through community group activities, a buddy system, and memory-making. Anticipatory guidance for the NICU stay was provided, including potential transitions to a subacute team, relocation to a separate area for less acute infants within our hospital, and even transfer to outlying lower level nurseries for convalescent care, if appropriate.

**Table 1.** PDSA Cycles Showing Date and Descriptions of PDSAs

PDSA Cycles	Description of PDSA
PDSA no. 1: May 2022	Targeted education of FCCCs
PDSA no. 2: October 2022	Initiation of care conferences
PDSA no. 3: December 2022	Creation of family pamphlet
PDSA no. 4: December 2022	Initiation of small baby rounds
PDSA no. 5: January 2023	Expansion of team to include family advisor and parent educator
PDSA no. 6: August 2023	Translation of written materials and conferences in Spanish
PSDA no. 7: February 2024	Expansion of team and transition of roles

After each conference, the team provides a survey to parents that has 2 components. The first section consists of 3 open-ended questions that allow families to provide constructive feedback regarding the content, key takeaways, and areas for improvement. The second section consists of 3 Likert scale questions asking whether the FCCC helped provide information on how parents could be involved in their infant’s care, whether they would recommend FCCC to other families, and whether they would like a second FCCC later in their stay. In response to their feedback, in December 2022 (PDSA no. 3), we created an informational pamphlet that illustrated the goals, topics, and photographs of the team. (**See figure 1, Supplemental Digital Content 2**, which displays a pamphlet handed out to families during the initial introduction of conferences demonstrating the goals, topics, and team involved in the FCCCs, https://links.lww.com/PQ9/A683.) In reviewing previous cycles, the team assessed that the timing of introducing the conferences to families was inconsistent. To aid in this, we introduced and scheduled conferences during small baby rounds, which occurred biweekly (PDSA no. 4). The multidisciplinary team expanded to include a nurse-parent educator and family advisor (PDSA no. 5, January 2023). As the patient population grew with more Spanish-speaking families, written materials were translated into Spanish, and an in-person interpreter provided translation to improve equity, inclusivity, and the expansion of the conferences (PDSA no. 6, August 2023). In February 2024, the team expanded to include another neonatology fellow to help introduce, schedule, facilitate, and sustain the FCCCs (PDSA no. 7).

## MEASURES

The primary outcome measure was the percentage of eligible infants whose families participated in FCCCs. A secondary outcome was the percentage of families attending their infant’s first NFC appointment after discharge. In addition, we tracked the percentage of timely conferences (FCCCs occurring between 7 and 28 d of life), the number of nontimely conferences between each timely FCCC, and the number of eligible infants between each FCCC who did not receive a conference. We also tracked the percentage of small babies who received the intervention before discharge and their responses to the postconference survey. The balancing measure was the number of patient encounters each team member conducted per month.

### Data Analysis

The team tracked all measures monthly on statistical process control (SPC) charts using QI Macros Software for Excel 2023 (KnowWare International, Inc., Denver, CO). We used established rules to differentiate between special and common cause variation. The team tracked PDSA cycles on control charts and analyzed the data to determine whether the interventions were successful or needed modification.

## RESULTS

**Fig. 2.**
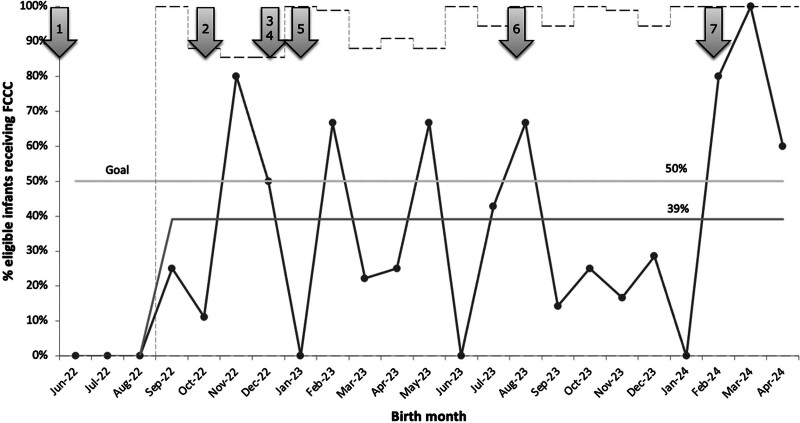
p-Chart shows the percentage of eligible infants whose families participated in an FCCC. Legend for arrows to corresponding PDSA cycles: 1, education of FCCC; 2, initiation of FCCCs; 3, creation of informational pamphlets for families; 4, initiation of small baby rounds; 5, expansion of team; 6, translation of FCCCs and informational materials to Spanish; 7, expansion of team and conceptualization of second FCCCs.

**Fig. 3.**
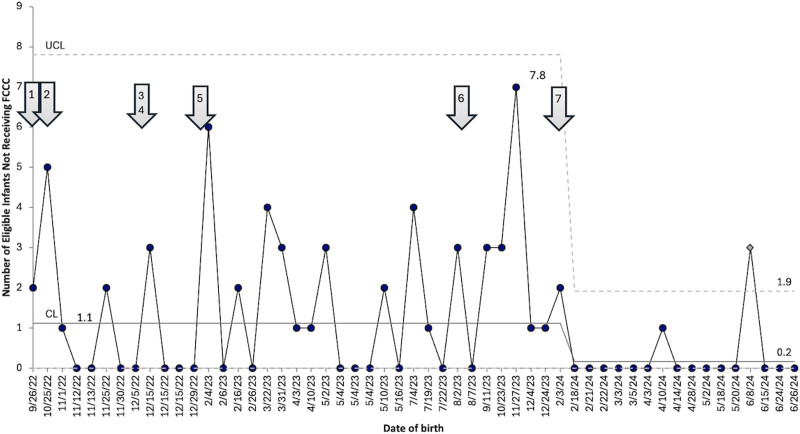
g-Chart demonstrating the number of eligible infants who did not receive an FCCC between each FCCC occurrence. The arrow highlightlighing when PDSA cycle number 7 began demonstrates a decrease from 1.1 eligible infants not receiving an FCCC to 0.2. Legend for arrows to corresponding PDSA cycles: 1, education of FCCC; 2, initiation of FCCCs; 3, creation of informational pamphlets for families; 4, initiation of small baby rounds; 5, expansion of team; 6, translation of FCCCs and informational materials to Spanish; 7, expansion of team and conceptualization of second FCCCs.

The primary outcome measure was the percentage of infants whose families participated in an FCCC and demonstrated special cause variation with a shift from 0% to 39% during an 18–month period (Fig. 2). We defined the baseline as the period before October 2022, because we implemented the first FCCC in that month. To track missed eligible infants, a g-chart (Fig. 3) was used to show the number of eligible infants who did not receive an FCCC (*y* axis) for each FCCC (*x* axis). The increased number of infants who did not receive an FCCC in November 2023 was due to several reasons such as patient acuity, aging out, maternal readmission, and a family whose primary language was not Spanish or English. However, the chart shows special cause variation beginning in February 2024, with a decrease from an average of 1.1 eligible infants not receiving a conference between each FCCC occurrence to 0.2, representing an 81% decrease. This signal in Figure 3 occurred after PDSA cycle number 7 when the scheduling workflow improved, and we had an additional facilitator for the conferences. This significant improvement has been sustained throughout the remainder of the project thus far.

**Fig. 4.**
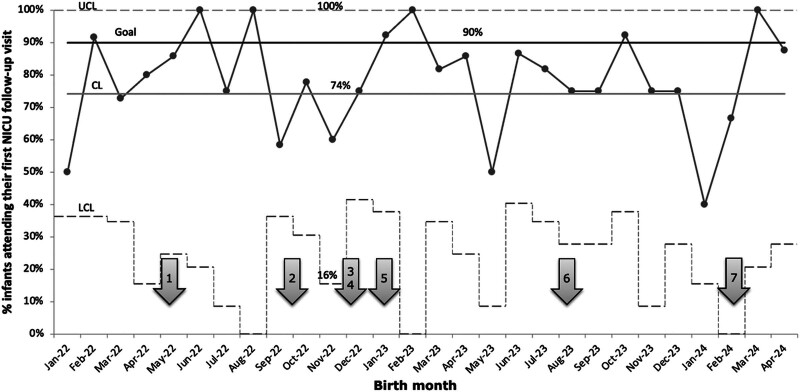
p-Chart demonstrates the percentage of small babies attending their first outpatient NFC follow-up clinic, with the centerline at 74% and the goal of 90%. Legend for arrows to corresponding PDSA cycles: 1, education of FCCC; 2, initiation of FCCCs; 3, creation of informational pamphlets for families; 4, initiation of small baby rounds; 5, expansion of team; 6, translation of FCCCs and informational materials to Spanish; 7, expansion of team and conceptualization of second FCCCs.

The second outcome measure was to increase the initial show rate of small babies to their first NFC appointment with the developmental pediatrician after discharge. From January 2022 to June 2023, the baseline show rate averaged 74%, depicted in a p-chart (Fig. 4). Since our FCCCs began in October 2022, we anticipated that follow-up rates would not start to reflect our quality improvement (QI) efforts until sometime after January 2023, as infants in the small baby program are seen at the NFC 6 weeks after discharge. Although we have not yet demonstrated special cause variation, our QI project has not negatively impacted outpatient NICU follow-up.

We intend to provide the FCCC at the appropriate time during the NICU stay, which we propose is between the first and fourth weeks of the infant’s life. To track the timeliness of FCCCs, we used an SPC g-chart to demonstrate the number of nontimely conferences (*y* axis) between each occurrence of a timely conference (*x* axis). Ideally, we would want there to be no nontimely conferences. (**See figure 2, Supplemental Digital Content 3**, which displays g-chart demonstrating the number of nontimely conferences between timely conferences. The chart showed special cause variation beginning in March 2024, with a decrease from an average of 0.9 nontimely conferences between FCCCs to 0. The arrow indicates when PDSA cycle number 7 occurred, which was when a dedicated team of people was in place to help introduce, facilitate, and schedule the FCCCs. https://links.lww.com/PQ9/A684.)

We focused several of our process measures on responses to the postconference surveys and tracked them using SPC p-charts. We did not include the p-charts in this article due to the similarity in results among the 3 questions tracked. Overall, 66% of families responded to the survey. All respondents (32/32 or 100%) agreed or strongly agreed that the conference provided information on how they can be involved in their infant’s care and would recommend FCCCs to other families. Nearly all (31/32 or 97%) agreed or strongly agreed that they would like a second FCCC closer to discharge. We did not observe special cause variation, which is likely due to the baseline responses already being 97% to 100%. Although we are unable to show a statistical improvement in our measure, this is indicative of the value families place on FCCCs. Comments from families as significant educational points included learning “how they can build a relationship with their child,” “what the going-home requirements were,” “how it is really beneficial to speak to everyone at once compared with intermittently in the baby’s room,” and how the “team feel” exhibited a new appreciation of the “unique role each provider plays.”

The team used a p-chart to demonstrate the percentage of small babies receiving an SSC episode before discharge. In our unit, 90% of small babies have an SSC episode before discharge. (**See figure 3, Supplemental Digital Content 4**, which displays p-chart demonstrating the percentage of small babies receiving an SSC episode before discharge. Ninety percent of small babies experience an SSC episode before discharge home. https://links.lww.com/PQ9/A685.) After we initiated the FCCCs, special cause variation occurred, with an increase to 97% beginning in December 2022, which coincided with the discharge of the first infants whose families attended an FCCC. We attributed this change to the education provided to families during the FCCC and the SSC initiative in our unit’s small baby program, which began in 2021.

**Fig. 5.**
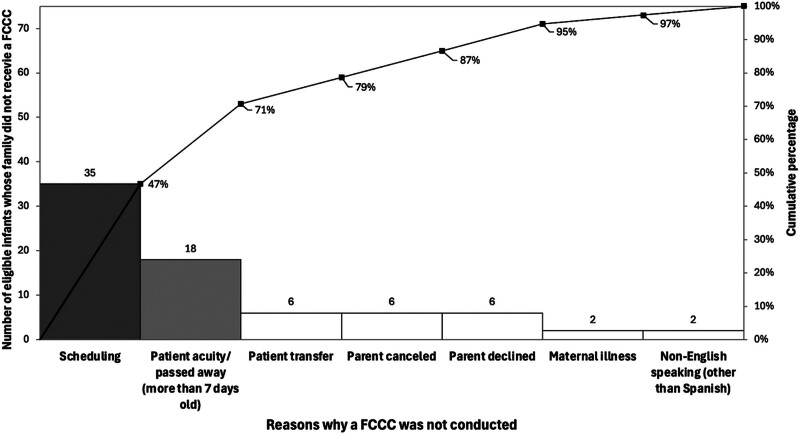
A Pareto chart demonstrates the reasons care conferences were not conducted, with scheduling challenges related to facilitator scheduling and patient acuity accounting for 71%.

As a balancing measure, the team tracked the patient care productivity of FCCC members (social work, occupational therapy, child life, and music therapy) to ensure that the conferences were not negatively affecting their direct patient care time. Using SPC c-charts, the authors observed that the number of patient encounters of each FCCC team member remained stable or even increased. (**See figures 4–7, Supplemental Digital Content 5**, which displays the c-chart that demonstrates the number of NICU social worker patient encounters. Legend for arrows to corresponding PDSA cycles: 1, education of FCCC; 2, initiation of FCCCs; 3, creation of informational pamphlets for families; 4, initiation of small baby rounds; 5, expansion of team; 6, translation of FCCCs and informational materials to Spanish; 7, expansion of team and conceptualization of second FCCCs, https://links.lww.com/PQ9/A686; the c-chart that demonstrates the number of patient encounters for NICU occupational therapists. https://links.lww.com/PQ9/A687; the c-chart that demonstrates the number of patient encounters for the NICU child life specialists. , https://links.lww.com/PQ9/A688; the c-chart that demonstrates the number of patient encounters for NICU music therapists. Between December 2022 and July 2023, the NICU music therapist was required to cover other units within the hospital, which is likely the reason for the decrease in encounters during that time. https://links.lww.com/PQ9/A689.) Noted in the figure descriptions were critical time elements independent of the FCCC initiative, which may have contributed to the increase or decrease in the number of patient encounters.

## DISCUSSION

During this initiative, the percentage of infants whose families participated in an FCCC increased from 0% to 39% (Fig. 2). To better conceptualize the impact of this QI work on families, this percentage represents more than 30 families who would benefit from an FCCC per year, based on an average of 70–100 small babies admitted annually. In contrast, no families previously had access to this resource. A systematic approach to scheduling conferences, which included timely follow-up with families during biweekly small baby rounds and adding valuable team members to help recruit families, contributed to this increase. The team held formal biannual and informal team meetings to reflect on previous conferences, discuss interim data, and identify changes to improve and standardize future FCCCs.

The team constructed a Pareto chart (Fig. 5) to identify barriers to FCCC completion. Scheduling issues accounted for 47% of the reasons why we did not conduct an FCCC, and patient acuity/mortality accounted for 25%. Less common reasons included patient transfers, parents canceling or declining, maternal illness, and lack of an in-person translator other than Spanish. To address scheduling issues, PDSA cycle number 7 focused on improving the workflow for educating families and scheduling FCCCs. Initially, only 1 team member approached families. As the FCCCs rolled out, the focus shifted to having a team designated to approach families and schedule conferences, which led to special cause variation in multiple measures, including a decrease in eligible infants not receiving an FCCC between each FCCC (Fig. 3) and improved timeliness of FCCC (**Supplemental Digital Content 3**, https://links.lww.com/PQ9/A684). Although there has not been enough time or data points to demonstrate a signal, following PDSA cycle number 7, the percentage of eligible infants receiving an FCCC has consistently been greater than 50%, and we anticipate this trend will continue to stay above our center line of 39% (Fig. 2). We plan to use future PDSA cycles to maintain this increase in percentage by focusing on scheduling FCCCs, offering conferences to families whose primary language is not English or Spanish, and emphasizing the option of attending virtual conferences if time and childcare are challenges.

Based on conversations during and after conferences, as well as responses to postconference surveys, families have expressed eagerness to actively participate in their infant’s care, understand their important role at the bedside, and appreciate the roles of specialists in optimizing their infant’s development. The period from birth to 3 years of age is crucial for brain development, as early experiences have a significant impact on outcomes.^9^ Exposures to stressors in the NICU are associated with regional alterations in brain structure and function.^10^ However, parent participation in the NICU, including providing 2-person care, breastfeeding, and SSC, is shown to decrease stress and pain experiences in this population. Additionally, brain development can be optimized by having parents engage with their newborn in the NICU.^2^ The team anticipates that increased family engagement will positively impact the infant’s length of stay and long-term developmental outcomes. As more families become engaged in caring for their infant from admission, they may feel more prepared for discharge and the transition home.

We did not observe a special cause variation in the show rate for the first NFC visit, possibly due to a high baseline percentage. We still consider this a priority for future directions of this project. We are planning to introduce a second FCCC closer to discharge to help families prepare for going home, understand follow-up care, and emphasize the importance of developmental therapies. We expect the second conference to have a significant impact on the NFC show rate in the future.

It is essential to conceptualize this initiative in a single, large regional perinatal center with dedicated NICU staff in the areas of family advisory, child life, and occupational and music therapy. Thus, the multidisciplinary nature of the FCCCs may be challenging in smaller NICUs with limited staffing and resources. Although the conference’s specifics may not be relevant to units that rarely care for extremely preterm infants, the concept of an FCCC benefits all patients admitted to the NICU, particularly those with anticipated lengthy admissions or need for multiple specialists and therapists. Additionally, the team educated families about SSC, 2-person care, and oral immunotherapy. Still, it did not track individual patient or family participation (looking at time spent or the number of episodes before and after we conducted an FCCC). Instead, we measured changes at the unit level over time and observed overall increases in these practices across the unit. Although multiple facets of the small baby program likely contributed to these improvements, we believe that family education through the FCCCs played a key role in our unit-wide success in family engagement.

To our knowledge, this project is the first QI initiative focused on implementing standardized FCCCs for NICU families with small babies. It was also the first to incorporate a multidisciplinary approach, including a family advisor, child life specialist, and music and occupational therapists. Our family advisor is a paid hospital employee who has personally experienced being a parent of a baby in our NICU and is involved in developing guidelines, policies, and QI initiatives to provide a family voice for our unit’s initiatives.

QI initiatives centered around families, education, and support depend upon the sustained efforts of a dedicated team of physicians, providers, nurses, and specialists. The sustainability may depend on providers’ ability to dedicate meaningful time, attention, and resources to such efforts. We aim to extend the information, education, and support provided during these conferences to all families with infants in the NICU who require prolonged care or have medical complexity.

## CONCLUSIONS

The team found that FCCCs improved family awareness of available NICU resources and family understanding of the value of their involvement in their infant’s well-being, comfort, and development. Further research is needed to better understand the relationships, behaviors, and emotions of families who have infants born extremely premature and how NICUs can better support and involve families during the initial weeks of life and throughout the NICU admission. The next steps of this project will focus on expanding the team further to engage more families in FCCCs and conceptualizing a second care conference focused on discharge readiness and the transition home.

## ACKNOWLEDGMENTS

The authors thank the families who participated in the care conferences. The authors would also like to acknowledge Cassandra Drees, MD, for her help in scheduling, facilitating, and sustaining the conferences; Tessa Banfield, LMSW; Rachel Dalberth, LMSW; Carla LeVant, LMSW; and Marnell Socci, RN, for their participation in several FCCCs; and Joan Merzbach for her assistance with data acquisition for the NFC. Dr. Mendoza would like to thank Jeff Meyers, MD, Rita Dadiz, DO, Patricia Chess, MD, Ponnila Marinescu, MD, and Carl D’Angio, MD, for their guidance on this initiative and review of the article.

## Supplementary Material


