# Supplementary material for: Improving Involvement of Families of Small Babies through Family Education, Family Integration, and Multidisciplinary Teamwork: A Quality Improvement Initiative
Source: Pediatr Qual Saf. 2025 Jul 30;10(4):e828. doi: 10.1097/pq9.0000000000000828 (PMC12309807; doi:10.1097/pq9.0000000000000828)
Supplement: Supplementary file 1 [file pqs-10-e828-s001.pdf]

Role	Aspects of Family Involvement Discussed During FCCCs
Facilitator (Neonatology Fellow/ Neonatologist)	Approach eligible families and schedule conferences<br>Summarize the discussion<br>Discuss the anticipated macrotransitions<br>Determine if any specific needs or milestones exist that the family would like to prioritize
Social Worker	Assist with transportation, temporary housing, parking<br>Introduce buddy program<br>Promote self-care
Child Life Specialist	Assess and support parent and sibling educational and psychosocial needs to promote an understanding of the patient's needs and capabilities, and to promote the use of positive coping strategies to enhance bonding<br>Provide non-pharmacologic pain management interventions to promote comfort during procedures<br>Support families in celebrating NICU-specific and traditional infant milestones and creating positive memories<br>Provide developmentally supportive interventions to promote patients' growth, development and comfort plus facilitates the provision of volunteer support when parents cannot be at bedside<br>Construct a developmentally appropriate support plan in collaboration with healthcare team members and patient's family
Music Therapist	Support neurosensory integration through positive auditory experiences (i.e., talking, reading, humming, singing)<br>Educate on auditory development and safe noise levels for infants<br>Educate about appropriate musical styles/complexities for each stage of infant development
Occupational Therapist	Support neurosensory development (i.e. eye protection, felt hearts, positive touch, standing transfers for skin-to-skin)<br>Promote joint, muscle and bone development through skin-to skin care and proper bundling and positioning<br>Promote oral immunotherapy for immune protection and pre-feeding enhancement<br>Provide techniques on two-person cares<br>Introduce role of physical therapy at 35 weeks

Lactation Medicine Specialist	Support parents' feeding goals<br>Help maximize milk supply<br>Support skin-to-skin care while pumping through a "snuggle and pump" garment
Nursing/ parent educator	Emphasize bedside care and involvement during care times<br>Enhance communication between family and developmental specialists, medical team and staff<br>Inquire about special considerations families desire (i.e. safeguarding the first feed, outfit or bath for the families, protecting story time, creating memories with infant's footprints)
Family Advisor	Support families through emphasis of self-care<br>Provide sessions on memory making through scrapbooking<br>Encourage a sense of community through group activities such as scrapbooking, family dinners and events such as "donuts with dad"<br>Provide peer one-on-one support to families

Team member roles and aspects of family involvement that are discussed during FCCCs
