# Supplementary material for: Improving Involvement of Families of Small Babies through Family Education, Family Integration, and Multidisciplinary Teamwork: A Quality Improvement Initiative
Source: Pediatr Qual Saf. 2025 Jul 30;10(4):e828. doi: 10.1097/pq9.0000000000000828 (PMC12309807; doi:10.1097/pq9.0000000000000828)
Supplement: Supplementary file 2 [file pqs-10-e828-s002.pdf]

QUESTIONS TO THINK ABOUT:

How can the NICU team help support you as a parent?
How can the NICU team help you meet your child's needs?
What is most important for you as a parent?

CONTACT US

Kris Nicole De Guzman, MD – NICU Fellow
Krisnicole_deguzman@urmc.rochester.edu

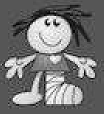

Golisano Children's Hospital
University of Rochester Medical Center
601 Elmwood Avenue #651
Rochester, New York 14642

888-794-URMC (888-794-8762)
www.golisano.urmc.edu

UR MEDICINE | GOLISANO CHILDREN'S HOSPITAL

MEDICINE of THE HIGHEST ORDER

YOUR BABY'S FAMILY-CENTERED CARE CONFERENCE

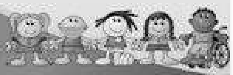

UR MEDICINE | GOLISANO CHILDREN'S HOSPITAL

MEDICINE of THE HIGHEST ORDER

WHAT IS A FAMILY-CENTERED CARE CONFERENCE?
Care conferences are a team approach to teach families on how to be actively involved in the care of your baby.

WHAT ARE THE GOALS OF THE FIRST CARE CONFERENCE?

- To meet the care team and learn about their roles
- Teach parents what they can do to support their baby in the NICU
- Help parents meet their baby's needs

WHAT TOPICS WILL BE DISCUSSED?

- Skin to skin care
- 2-person cares
- Bedside care
- Breastmilk supply
- Oral care
- Transitions in the NICU
- Infant development and support
- Family education and support
- Family experience

WHO WILL BE THERE?

Social Work

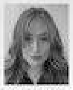
Rachel Dalberth, LMSW

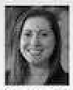
Molly Roberts, LCSW

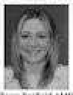
Tessa Banfield, LMSW

Occupational Therapy

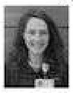
Laura Koegst, OT

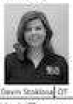
Devin Stoklosa, OT

Child Life

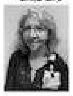
Chris Tryon, CCLS

Music Therapy

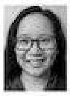
Elaine Kong, MT-BC

Lactation/Breastfeeding Medicine

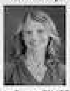
Lynsey Garver, RN, IBCLC

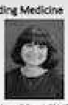
Marnell Sopci, RN, IBCLC

Assistant Nurse Manager / Small Baby Program

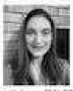
Hyacinth Lewis, BSN, CCRN-N

Parent Educator

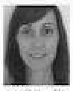
Jean Shilley, RN

Family Advisor

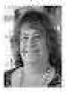
Tricia White

NICU Fellow

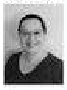
CJ Drees, MD

NICU Fellow

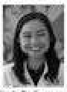
Nicole De Guzman, MD

NICU Attending

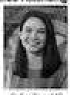
Colby Day, MD

*Your baby's nurse, primary team, maternal-fetal medicine doctor may also be able to join.
