# Supplementary figures and images for: Improving Involvement of Families of Small Babies through Family Education, Family Integration, and Multidisciplinary Teamwork: A Quality Improvement Initiative
Source: Pediatr Qual Saf. 2025 Jul 30;10(4):e828. doi: 10.1097/pq9.0000000000000828 (PMC12309807; doi:10.1097/pq9.0000000000000828)

gChart of Number of Non-timely Conferences Between Timely Conferences

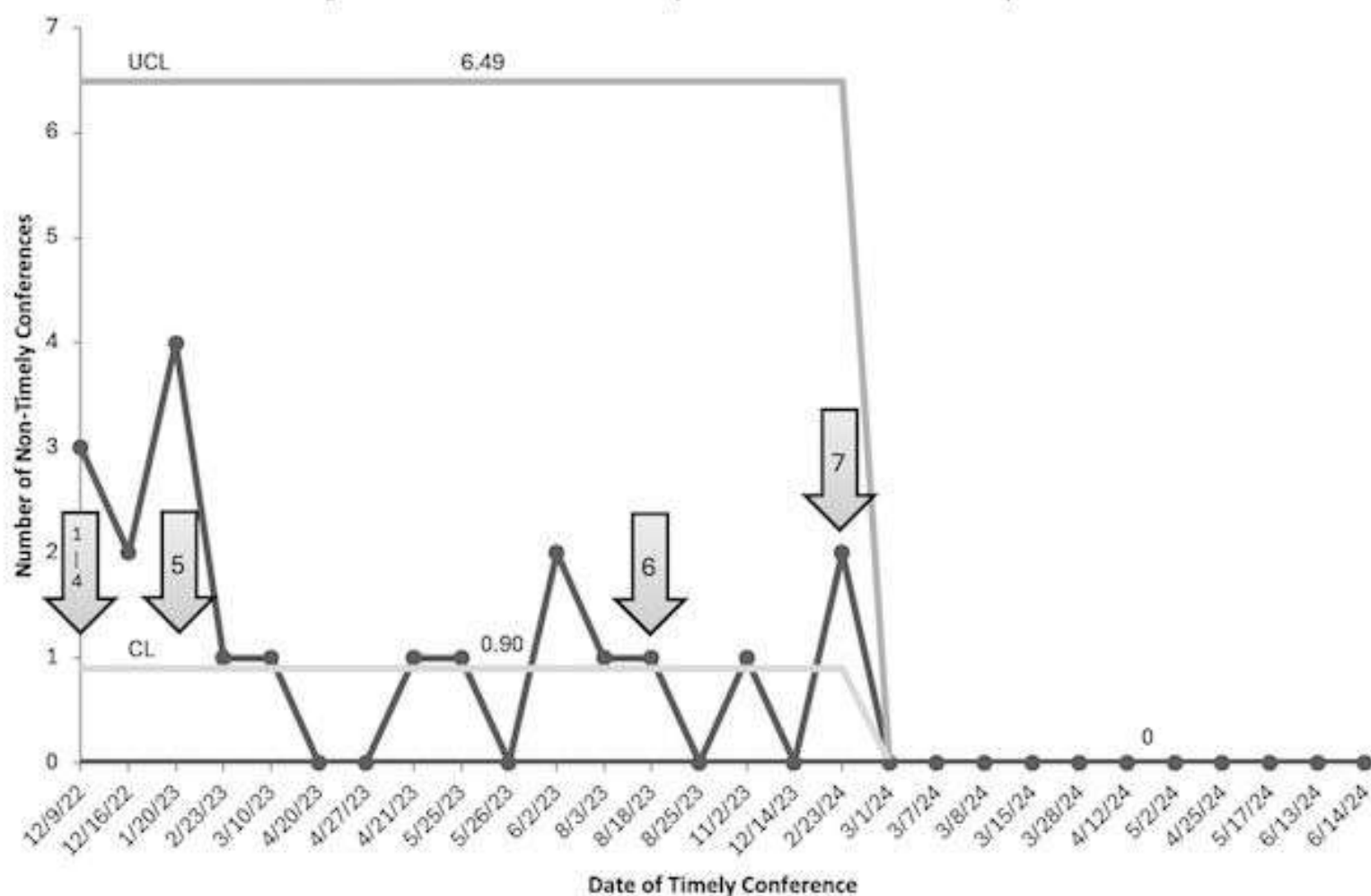

Supplement: Supplementary file 3 [file pqs-10-e828-s003.pdf]

% Small babies with atleast 1 skin to skin episode p-Chart  
n=156

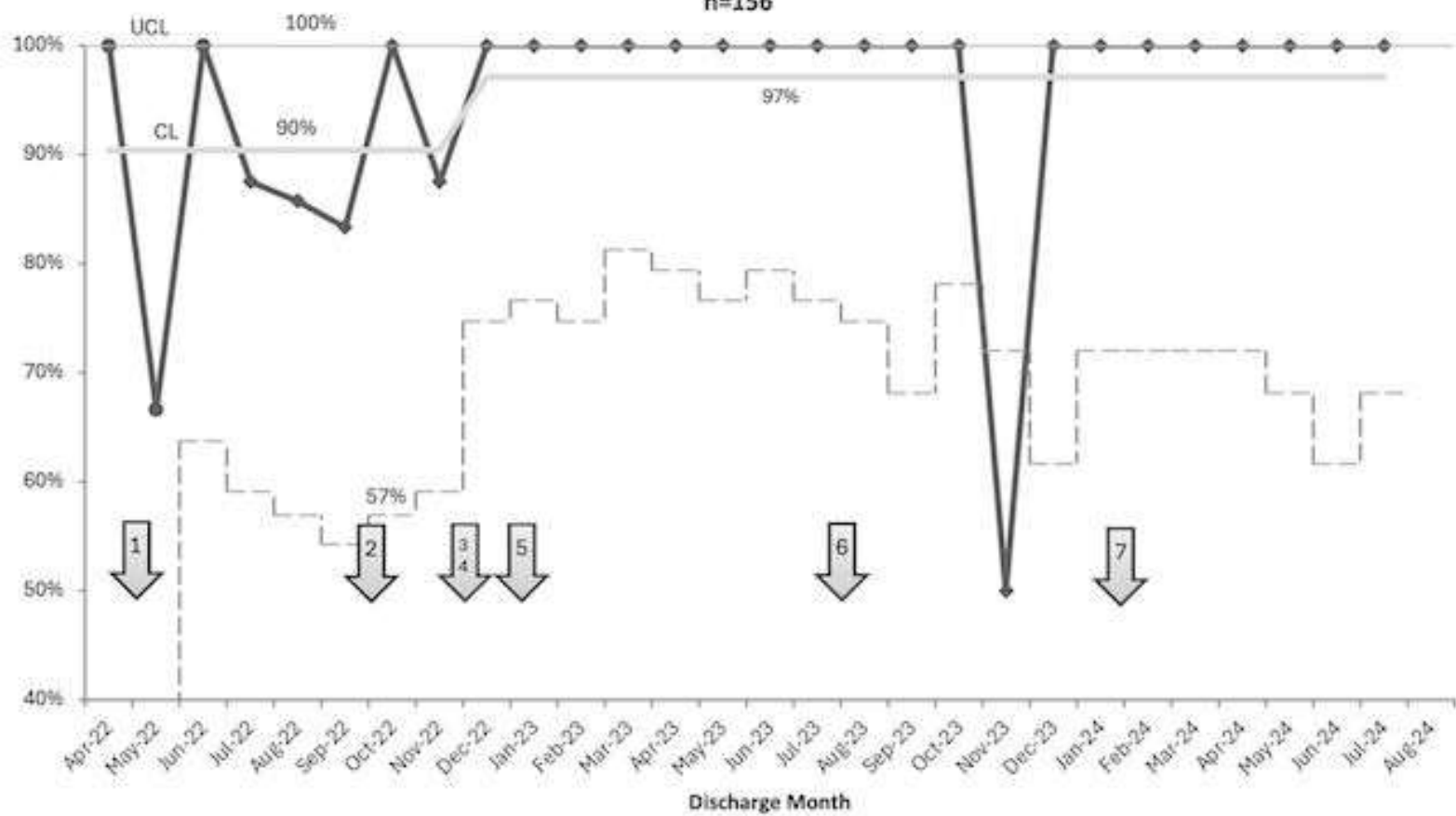

Supplement: Supplementary file 4 [file pqs-10-e828-s004.pdf]

NICU Social Workers

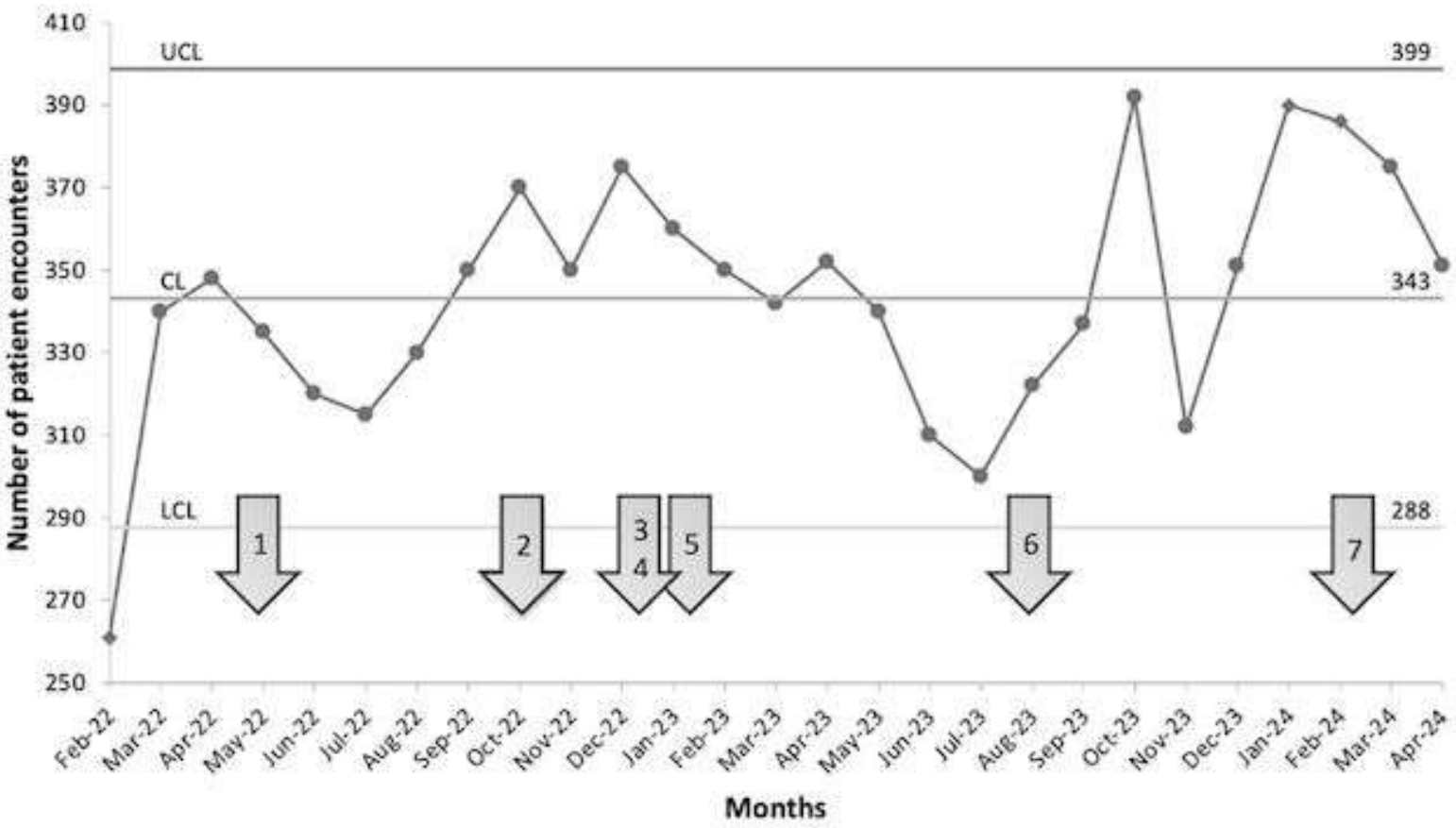

Supplement: Supplementary file 5 [file pqs-10-e828-s005.pdf]

NICU Occupational Therapists

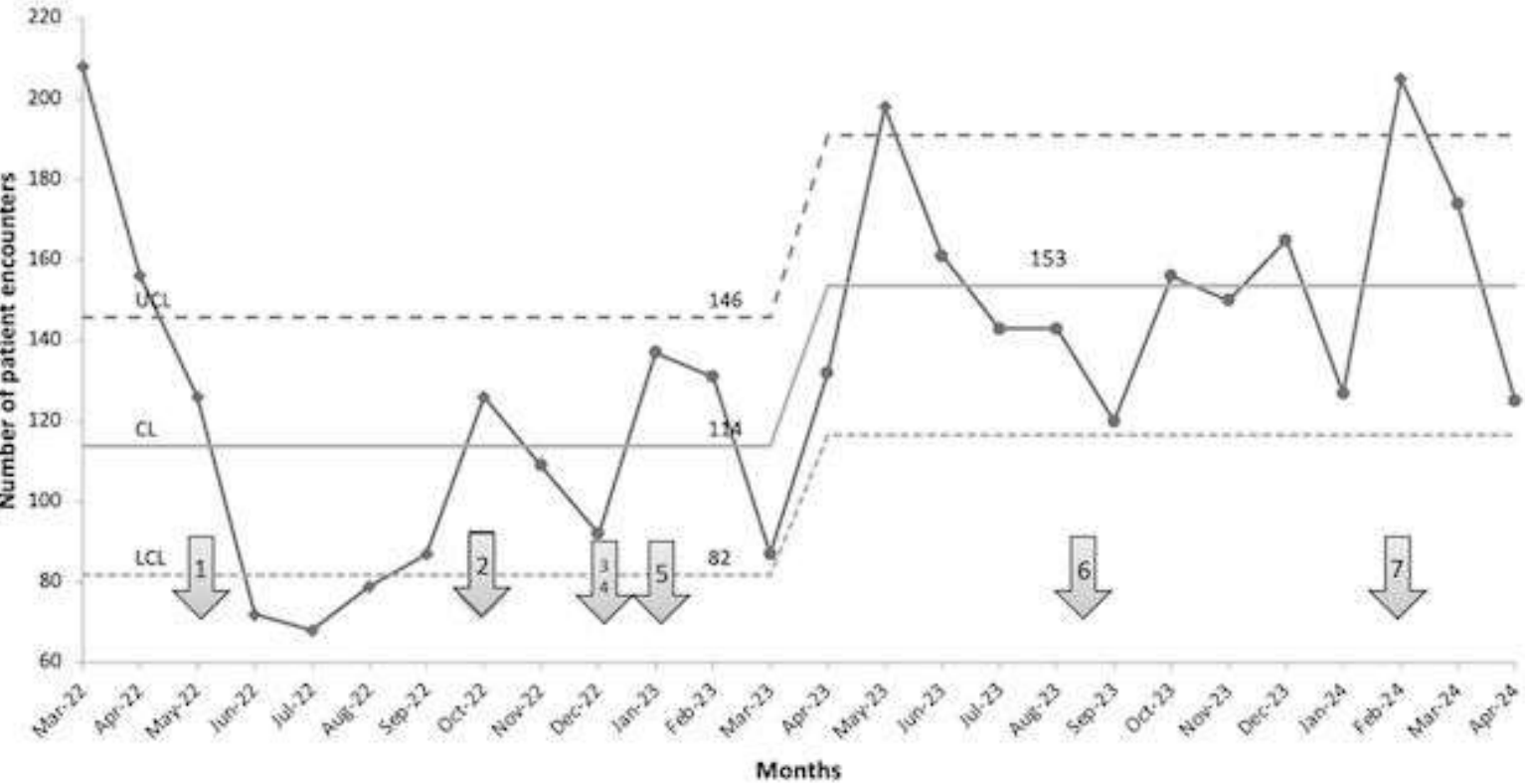

Supplement: Supplementary file 6 [file pqs-10-e828-s006.pdf]

NICU Child Life Specialists

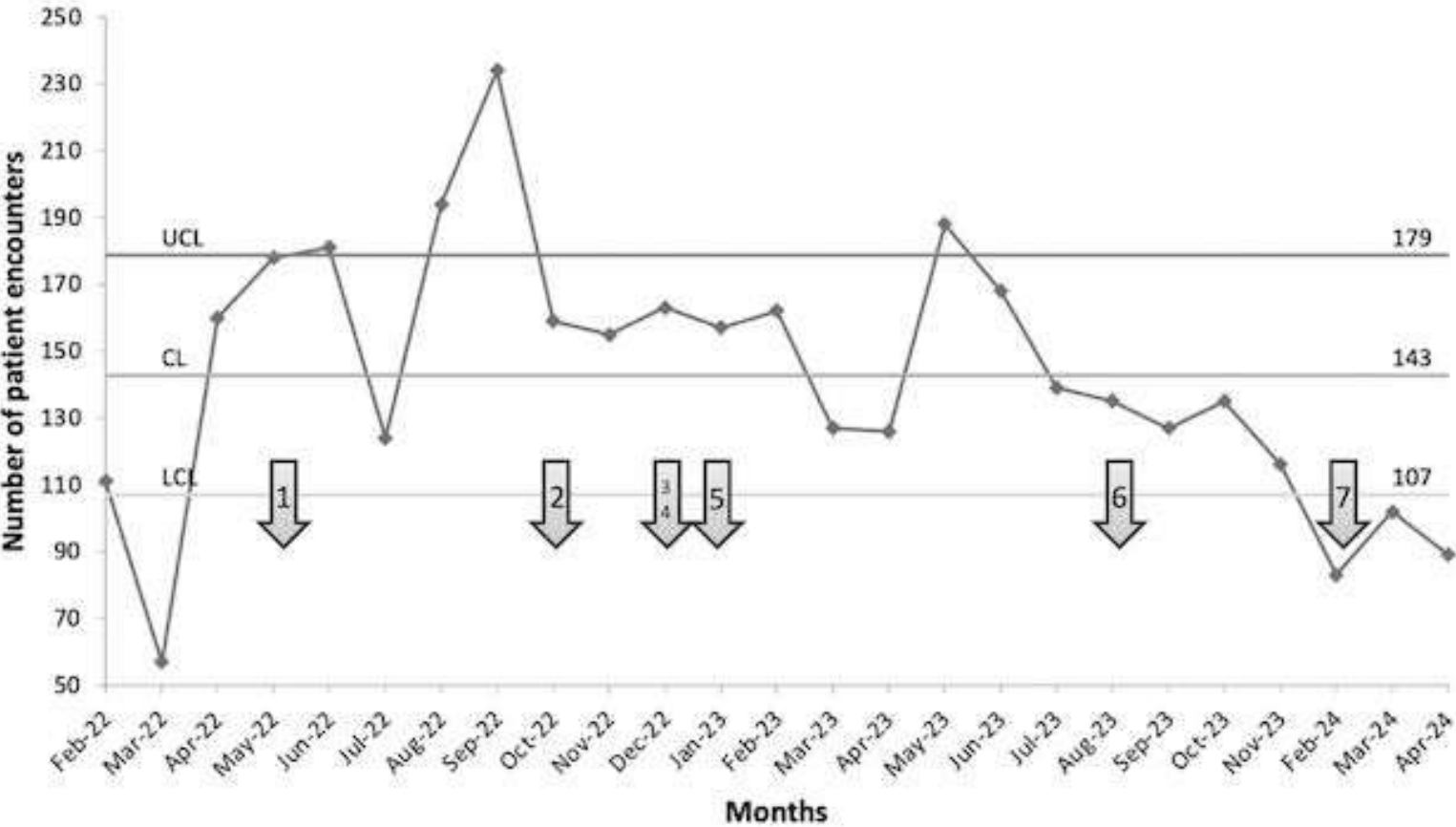

Supplement: Supplementary file 7 [file pqs-10-e828-s007.pdf]

NICU Music Therapist

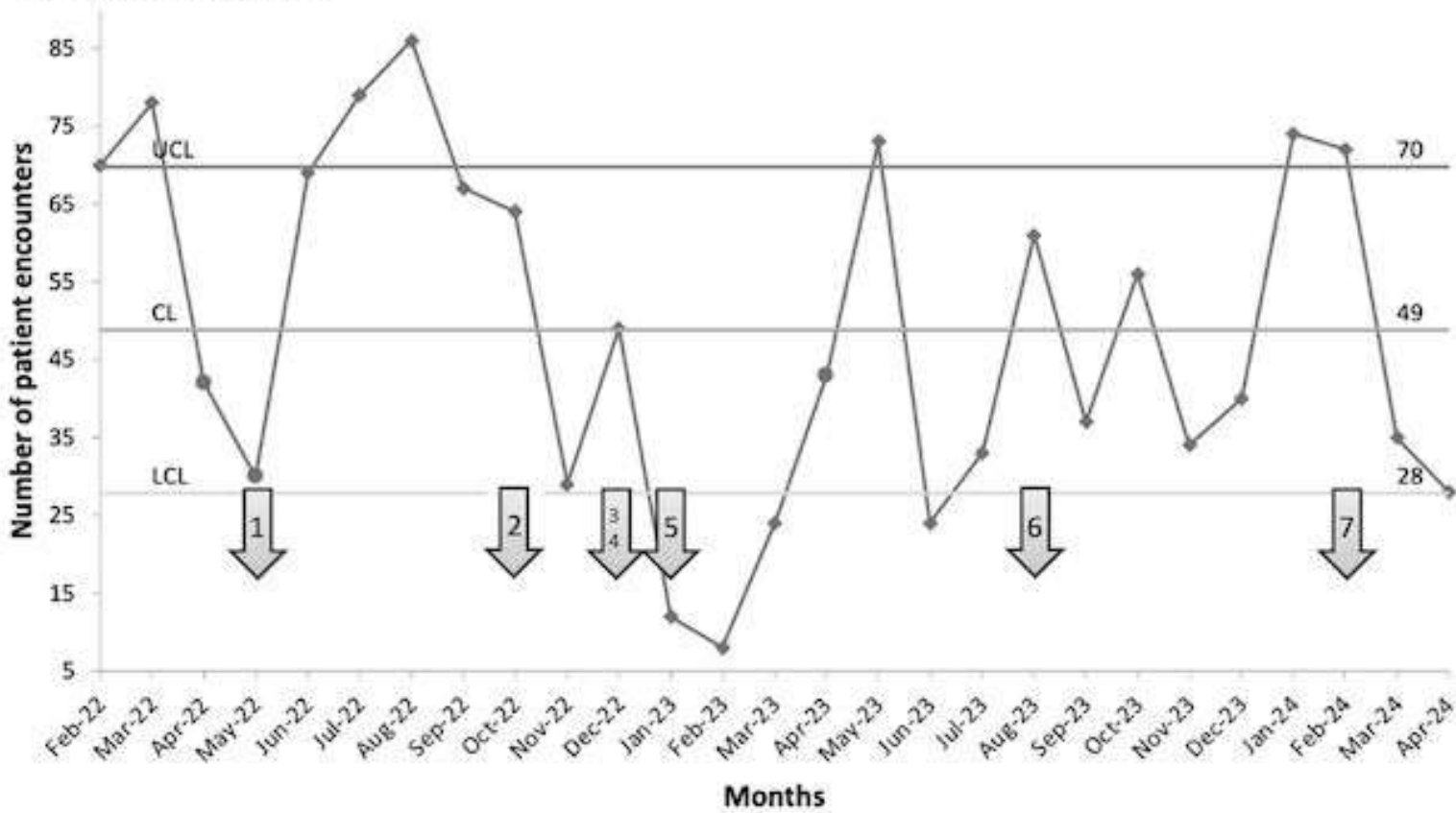

Supplement: Supplementary file 8 [file pqs-10-e828-s008.pdf]
